# CG6015 controls spermatogonia transit-amplifying divisions by epidermal growth factor receptor signaling in *Drosophila* testes

**DOI:** 10.1038/s41419-021-03783-9

**Published:** 2021-05-14

**Authors:** Jun Yu, Qianwen Zheng, Zhiran Li, Yunhao Wu, Yangbo Fu, Xiaolong Wu, Dengfeng Lin, Cong Shen, Bo Zheng, Fei Sun

**Affiliations:** 1 Institute of Reproductive Medicine, School of Medicine, Nantong University, Nantong, China; 2 Department of Gynecology, the Affiliated Hospital of Jiangsu University, Jiangsu University, Zhenjiang, China; 3 State Key Laboratory of Reproductive Medicine, Center for Reproduction and Genetics, Suzhou Municipal Hospital, The Affiliated Suzhou Hospital of Nanjing Medical University, Gusu School, Nanjing Medical University, Suzhou, China

**Keywords:** Cell proliferation, Differentiation

## Abstract

Spermatogonia transit-amplifying (TA) divisions are crucial for the differentiation of germline stem cell daughters. However, the underlying mechanism is largely unknown. In the present study, we demonstrated that CG6015 was essential for spermatogonia TA-divisions and elongated spermatozoon development in *Drosophila* melanogaster. Spermatogonia deficient in CG6015 inhibited germline differentiation leading to the accumulation of undifferentiated cell populations. Transcriptome profiling using RNA sequencing indicated that CG6015 was involved in spermatogenesis, spermatid differentiation, and metabolic processes. Gene Set Enrichment Analysis (GSEA) revealed the relationship between CG6015 and the epidermal growth factor receptor (EGFR) signaling pathway. Unexpectedly, we discovered that phosphorylated extracellular regulated kinase (dpERK) signals were activated in germline stem cell (GSC)-like cells after reduction of CG6015 in spermatogonia. Moreover, Downstream of raf1 (Dsor1), a key downstream target of EGFR, mimicked the phenotype of CG6015, and germline dpERK signals were activated in spermatogonia of *Dsor1* RNAi testes. Together, these findings revealed a potential regulatory mechanism of CG6015 via EGFR signaling during spermatogonia TA-divisions in *Drosophila* testes.

## Introduction

The balance between proliferation and differentiation is crucial for germline homeostasis and is tightly controlled by the stem cell niche and spermatogonia transit-amplifying (TA) divisions^1^. In *Drosophila* melanogaster testes, the germline differentiation program must coordinate with germline stem cells (GSCs) self-renewal, and meiosis must take place at the appropriate time during spermatogonia TA-divisions^2^. However, the mechanisms regulating the homeostasis between proliferation and differentiation during spermatogonia TA-divisions are not fully understood.

Regulation of germ cell differentiation has been implicated to involve a number of classic signaling pathways. For instance, germ cell differentiation is repressed by Bone Morphogenetic Protein (BMP) signaling. Decapentaplegic (Dpp) and Glass Bottom Boat (Gbb), which are secreted by somatic hub cells and cyst cells, activate BMP signaling in GSCs and inhibit *bam* expression to prevent GSC differentiation^3,4^. The Bam protein is critical for the switch from proliferation to meiotic differentiation^5^. Importantly, an aberrant reduction in the Bam level arrests the differentiation pathway and generates extra GSC-like cells^6^.

The Epidermal Growth Factor Receptor (EGFR) signaling pathway is involved in cancer, proliferation, and cell fate determination^1,7,8^. Interestingly, aberrant EGFR signaling results in abnormal organ formation and tumorigenesis^9,10^. EGFR signaling is also essential for the self-renewal of follicle stem cells (FSCs) through the LKB1-AMPK pathway, while Notch signaling promotes differentiation of the prefollicle cells in the *Drosophila* ovary^11,12^. Groucho could be phosphorylated by EGFR signaling and inhibits Notch to trigger FSCs differentiation^13^. Meanwhile, extracellular regulated kinase (ERK) regulates high mitochondrial membrane potential, and the phosphorylated form of ERK (dpERK) is increased in *drp1* mutant posterior follicle cells in the *Drosophila* ovary^14^. Moreover, multiple studies have demonstrated that EGFR in somatic cells is critically important for the balance of self-renewal and differentiation in *Drosophila* testes^1,15,16^. Germline specific ligand-EGF catalyzes the activation of the EGFR signaling pathway by somatic receptors, leading to the aggregation of adapter protein like Grb2 and the guanine exchange factor Sos. Membrane associated Ras protein is triggered by Sos, and thereby activates the phosphorylation of MAPK cascades, which are consists of three kinases including Raf, MEK (Downstream of raf1, Dsor1) and ERK (rolled, rl). Ras phosphorylates MEK, and then ERK is dually phosphorylated by MEK. dpERK in turn phosphorylates and modulates a wide range of substrates both in cytoplasm and nucleus of cells^17–19^. Somatic EGFR plays key roles in the enclosure of GSCs and triggers the early steps of germline differentiation in *Drosophila* testes^20^. Downregulation of EGFR in cyst cells affects synchronized spermatogonia TA-divisions and the localization of Armadillo on the cyst membrane, while somatic EGFR activation is not required to induce Bam expression^21^.

In the present study, we investigated the transition from proliferation to differentiation during spermatogonia TA-divisions in *Drosophila* testes. Previously, a large-scale RNA interference (RNAi) screen identified a series of genes required for GSC maintenance^22^, among which a novel GSC regulator, CG6015, which might assist in the mRNA splicing process. The results of the present study show that CG6015 is a key module for spermatogonia TA-divisions, and that it regulates germline differentiation via germline dpERK signals. Furthermore, we performed a transcriptome analysis of CG6015-mediated regulatory network using RNA sequencing (RNA-seq) and revealed the role of EGFR signaling in spermatogonia TA-divisions in *Drosophila* testes.

## Results

### Spermatogonia derived CG6015 was necessary for cell differentiation and elongated spermatozoon development

**Fig. 1:**
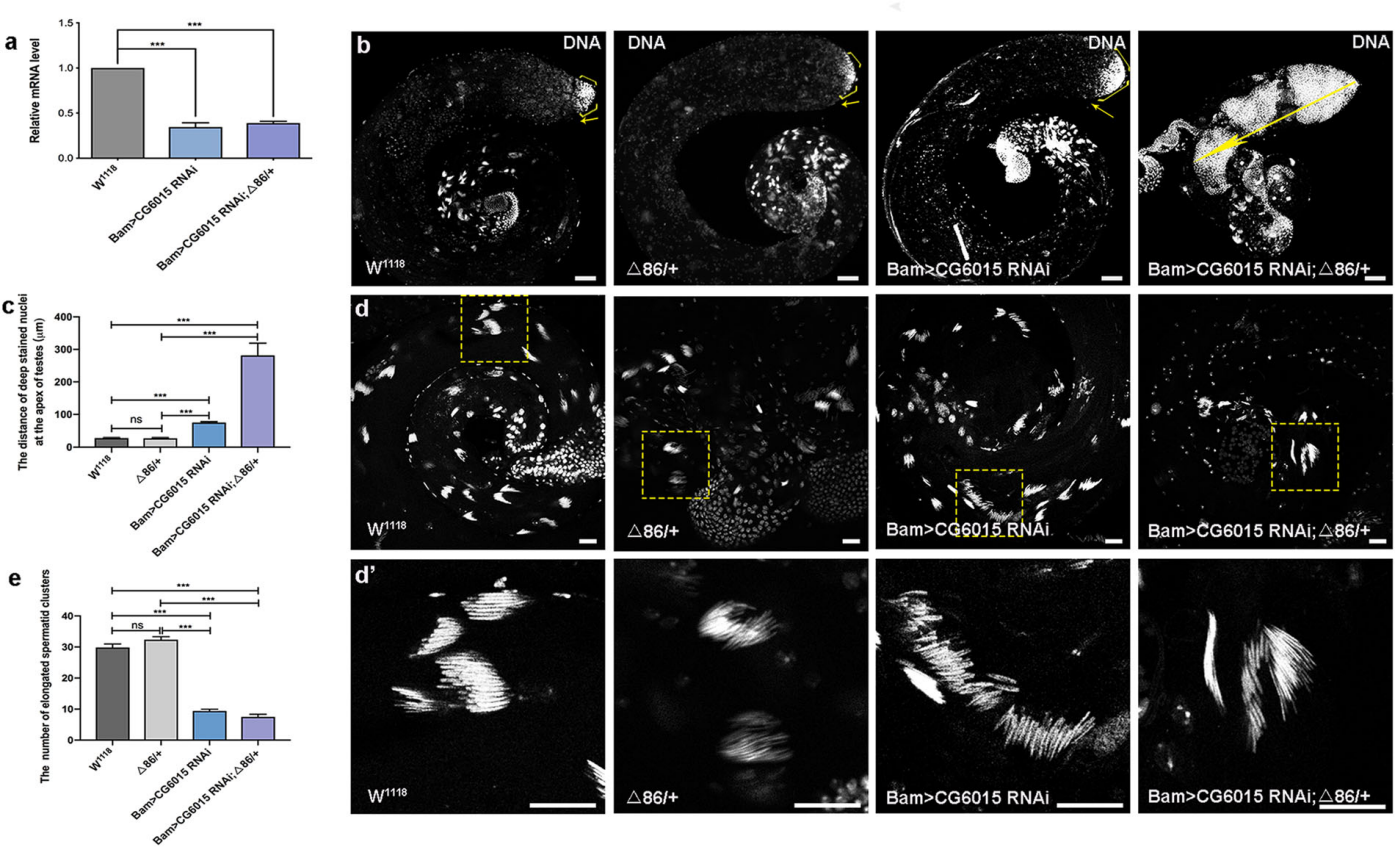
Reduction of CG6015 in spermatogonia led to accumulation of undifferentiated cells and elongated spermatozoon defects. **a** Relative mRNA level of *CG6015* in the W^1118^, Bam>*CG6015* RNAi and Bam>*CG6015* RNAi; Δ86/+ testes. **b** Whole mount DNA staining of the W^1118^, Δ86/+ , Bam>*CG6015* RNAi and Bam>*CG6015* RNAi; Δ86/+ testes. Yellow arrows represent undifferentiated cells at the apex of the testis. **c** The distance of deep stained nuclei at the apex of W^1118^ (*n* = 50), Δ86/+ (*n* = 27), Bam>*CG6015* RNAi (*n* = 36) and Bam>*CG6015* RNAi; Δ86/+ (*n* = 16) testes. **d** Elongated spermatid clusters at the tail of testes. **d’** Enlargements of corresponding elongated spermatid clusters. **e** The number of elongated spermatid clusters in W^1118^ (*n* = 44), Δ86/+ (*n* = 25), Bam>*CG6015* RNAi (*n* = 33) and Bam>*CG6015* RNAi; Δ86/+ (*n* = 10) testes. DNA was stained with Hoechst (blue). ***P < 0.001, ns. not significant. Scale bar: 50 μm for **b**; 20 μm for **d** and **d’**.

To investigate the role of CG6015 in spermatogonia in *Drosophila* testes, we employed the upstream activating sequence (UAS)/Gal4 system to manipulate the levels of CG6015 during spermatogonia TA-divisions, which was driven by Bam-Gal4. Quantitative real-time reverse transcription PCR (qRT-PCR) was used to verify the knockdown efficiency and reduced relative mRNA level of *CG6015* in *CG6015* RNAi testes (Fig. 1a). DNA staining could mark the early-stage nuclei at the apex of the testis and the nuclei of clusters of elongated spermatozoa at the tail of the testis^23,24^. In this study, we found that early-stage cells accumulated and the distance of deeply stained nuclei increased at the apex of Bam>*CG6015* RNAi testes, indicating that the differentiation process was inhibited after reducing the *CG6015* level in spermatogonia (Fig. 1b–c). Previous reports have shown that the Bam protein, which acts as a key differentiation factor, is essential for germ cell differentiation in both testes and ovaries in *Drosophila*^25–28^. Δ86/+, a heterozygous *bam*^Δ86^ null allele^29^, has been used to enhance germ cell differentiation phenotype in *Drosophila* testes^25^. Here, differentiation defects were further enhanced by heterozygous mutation of *bam* (Δ86/+) in Bam>*CG6015* RNAi testes (Fig. 1b–c).

Meanwhile, we also observed that the structure of clusters of elongated spermatids was loose and the number of nuclei of the clusters of elongated spermatids was reduced at the tail of *CG6015* RNAi testis when compared with the controls (Fig. 1d–e). Our results demonstrated that the number of clusters of elongated spermatozoa was dramatically decreased in Bam>*CG6015* RNAi; Δ86/+ testes (Fig. 1d–e). Taken together, our data indicated that CG6015 participates in differentiation and affects elongated spermatozoon formation in *Drosophila* testes.

### CG6015 was required for spermatogonia TA-divisions

**Fig. 2:**
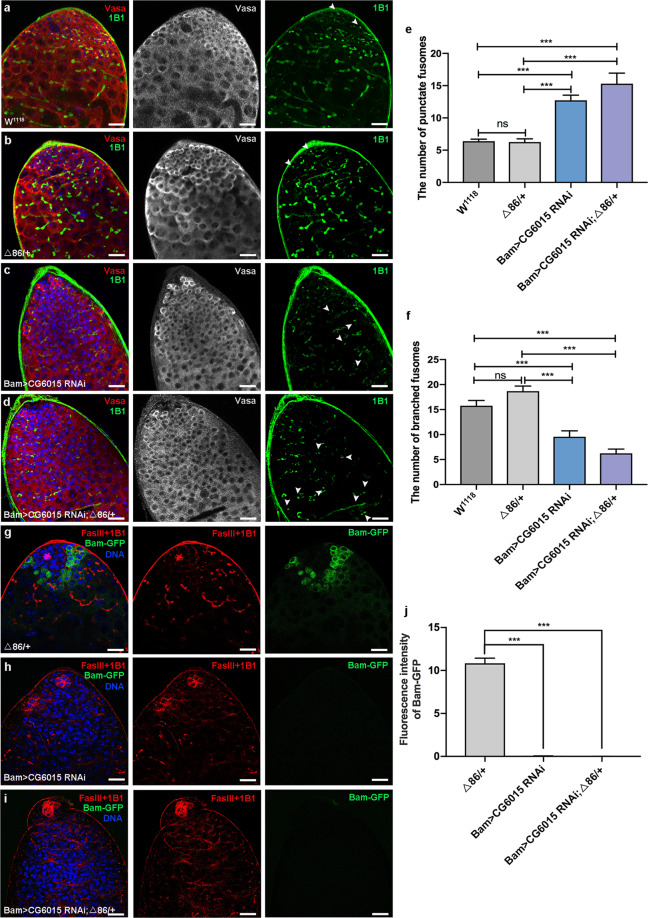
Reduction of CG6015 in spermatogonia restrained germline differentiation. **a–d** Immunostaining of Vasa (red) and 1B1 (green) at the apex of W^1118^, Δ86/+, Bam>*CG6015* RNAi and Bam>*CG6015* RNAi; Δ86/+ testes. **e** The number of punctate fusomes (also defined as ‘unbranched fusomes’ among germ cells) at the apex of W^1118^ (*n* = 13), Δ86/+ (*n* = 12), Bam>*CG6015* RNAi (*n* = 11) and Bam>*CG6015* RNAi; Δ86/+ (*n* = 13) testes. **f** The number of branched fusomes at the apex of W^1118^ (*n* = 13), Δ86/+ (*n* = 12), Bam>*CG6015* RNAi (*n* = 11) and Bam>*CG6015* RNAi; Δ86/+ (*n* = 13) testes. **g–i** Immunostaining of FasIII (red), 1B1 (red) and Bam-GFP (green) at the apex of Δ86/+ , Bam>*CG6015* RNAi and Bam>*CG6015* RNAi; Δ86/+ testes. **j** Fluorescence intensity of Bam-GFP at the apex of Δ86/+ (*n* = 15), Bam>*CG6015* RNAi (*n* = 3) and Bam>*CG6015* RNAi; Δ86/+ (*n* = 3) testes. DNA was stained with Hoechst (blue). ***P < 0.001, ns. not significant. Scale bars: 20 µm.

To further explore the roles of CG6015 during germline differentiation, we analyzed morphological changes of fusomes using immunofluorescence staining. Fluorescence tomography imaging has shown that fusomes, which are connections and special structures among germ cells, undergo dynamic changes from a punctate to a branched morphology during germline differentiation^30,31^. In this study, we observed that undifferentiated germ cells, which were marked by DNA and Vasa, accumulated in Bam>*CG6015* RNAi testes (Fig. 2a–c). Moreover, the phenotype of germline differentiation defects could be enhanced in the background of heterozygous mutation of *bam* in Bam>*CG6015* RNAi testes (Fig. 2a–d). Meanwhile, the number of punctate fusomes increased and the number of branched fusomes decreased in Bam>*CG6015* RNAi and Bam>*CG6015* RNAi; Δ86/+ testes when compared with control testes (Fig. 2e–f).

To further analyze the differentiation situation of germ cells, we examined Bam-green fluorescent protein (GFP) signals, a recombination protein marks endogenous Bam expression level^32^, in Δ86/+, Bam>*CG6015* RNAi, and Bam>*CG6015* RNAi; Δ86/+ testes. In Δ86/+ testes, a high level of Bam-GFP signals was detected among differentiated spermatogonia (Figs. 2g and 2j) as previous reported^25^. However, the Bam-GFP signal could be almost undetectable in Bam>*CG6015* RNAi or Bam>*CG6015* RNAi; Δ86/+ testes, which indicated that the germline differentiation process was inhibited by the loss of CG6015 in spermatogonia (Fig. 2g–j).

### Spermatogonia derived CG6015 non-autonomously contributed to the cyst cell differentiation

Previous studies have shown that GSC regulators could maintain the testicular structure and cyst cell survival^22^. We next investigated the maintenance of cyst cells using cyst cell markers (Zfh1 and Eya) under conditions of loss of CG6015 in spermatogonia. In control testes, Zfh1 could label cyst stem cells (CySCs) and the early stage of cyst cells, while Eya was the marker of mature cyst cells^31,33^. The decay of Zfh1 is necessary for promoting the differentiation of cyst cell lineage^33^. Unexpectedly, in both Bam>*CG6015* RNAi and Bam>*CG6015* RNAi; Δ86/+ testes, we found that the number of early-stage cyst cells and mature cyst cells were significantly increased when compared with control testes (Supplementary Fig. S1a–f). More importantly, the number of Eya/Zfh1 double positive cells was also obviously increased in both Bam>*CG6015* RNAi and Bam>*CG6015* RNAi; Δ86/+ testes (Supplementary Fig. S1g). These data indicated that reduction of CG6015 in spermatogonia could also disrupt the differentiation of cyst cells via non-autonomous effects.

### *CG6015* RNAi in spermatogonia caused abnormal proliferation

**Fig. 3:**
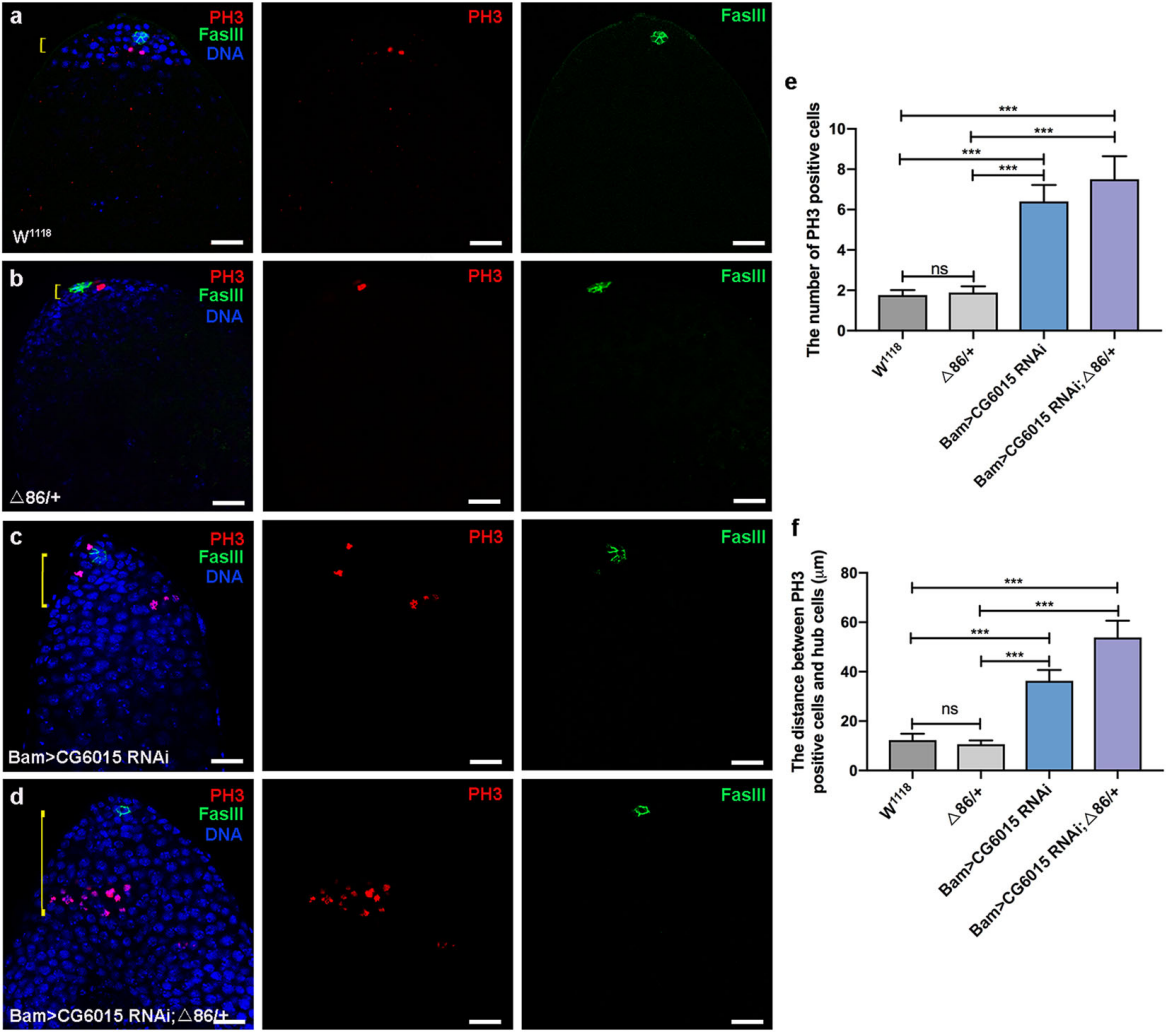
Reduction of CG6015 in spermatogonia increased proliferation in *Drosophila* testes. **a–d** Immunostaining of PH3 (red) and FasIII (green) at the apex of W^1118^, Δ86/+ , Bam>*CG6015* RNAi and Bam>*CG6015* RNAi; Δ86/+ testes. DNA was stained with Hoechst (blue). Yellow lines represented the distance between PH3 positive cells and hub cells. **e** The number of PH3 positive cells in W^1118^ (*n* = 12), Δ86/+ (*n* = 9), Bam>*CG6015* RNAi (*n* = 10) and Bam>*CG6015* RNAi; Δ86/+ (*n* = 10) testes. **f** The distance between PH3 positive cells and hub cells in W^1118^ (*n* = 12), Δ86/+ (*n* = 9), Bam>*CG6015* RNAi (*n* = 11) and Bam>*CG6015* RNAi; Δ86/+ (*n* = 10) testes. ***P < 0.001, ns. not significant. Scale bars: 20 µm.

Early stages of germ cells could be maintained and controlled by hub signals^34^. CySCs play instructive roles for the proper onset of the germline differentiation, and loss of CySCs would lead to accumulation of GSC-like cells and failure to enter the TA process in *Drosophila* testes^35^. Although CySCs and mature cyst cells provide differentiation microenvironments, we found that reduction of CG6015 in spermatogonia directly led to differentiation defects without normal hub signals, and undifferentiated GSC-like cells, which were far away from hub cells, also acquired self-proliferation ability (Fig. 3a–d). Moreover, the number of PH3-positive cells and the distance between PH3-positive and hub cells were increased in both Bam>*CG6015* RNAi and Bam>*CG6015* RNAi; Δ86/+ testes when compared with those of control testes (Fig. 3e–f).

### Transcriptome analysis of CG6015 mediated regulatory network

**Fig. 4:**
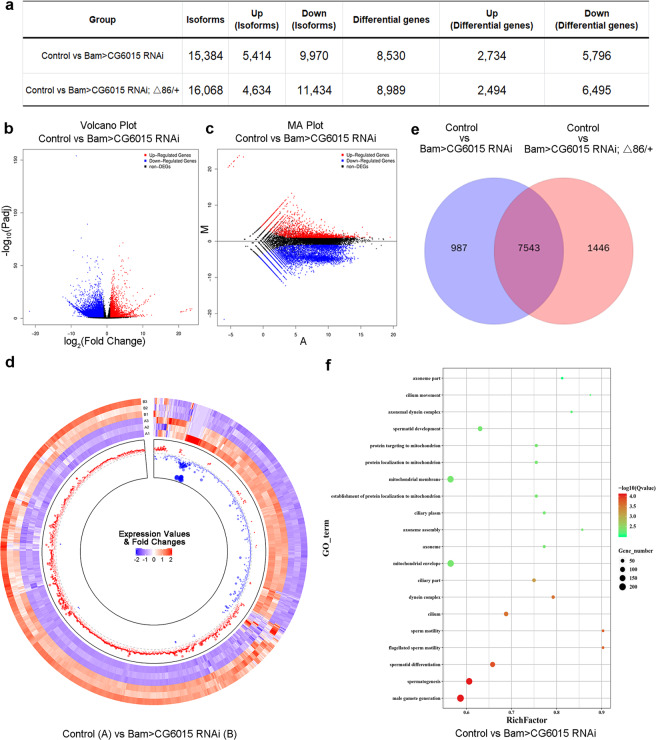
Reduction of CG6015 in spermatogonia induced transcript alterations as assessed using RNA-seq analysis. **a** Number of isoforms and genes identified by the RNA-seq in testes. **b** Volcano plots based on –log_10_Padj and log_2_FC from the comparison of the control (W^1118^) and Bam>*CG6015* RNAi groups. **c** MA plots from the comparison of the control (W^1118^) and Bam>*CG6015* RNAi groups. **d** Circular heatmap of differentially expressed genes from the comparison of the control (W^1118^) and Bam>*CG6015* RNAi groups. The outer and inner tracks represent the expression values and fold changes, respectively. For both the expression value and fold change, blue and red scales represented low and high expression correspondingly. **e** Venn diagrams comparing the sets of differentially expressed genes. **f** GO analysis of the sets of differentially expressed genes from the comparison of the control (W^1118^) and Bam>*CG6015* RNAi groups.

To gain better understandings of the regulatory network mediated by CG6015 in testes, we next carried out RNA-seq analysis of control, Bam>*CG6015* RNAi, and Bam>*CG6015* RNAi; Δ86/+ testes. In total (Supplementary Tables S1–S2), 42895 isoforms and 18456 genes were detected in the transcriptional profiles, and differentially expressed isoforms and genes were further analyzed [false discovery rate (FDR) < 0.05; log_2_ fold-change (FC) > 1 or log_2_FC < −1]. Among them (Fig. 4a and Supplementary Tables S3–S4), we identified 15384 isoforms (5414 upregulated isoforms and 9970 downregulated isoforms) corresponding to 8530 differentially expressed genes (2734 upregulated genes and 5796 downregulated genes) between the control and Bam>*CG6015* RNAi testes. Moreover, we analyzed differentially expressed isoforms and genes between the control and Bam>*CG6015* RNAi; Δ86/+ testes, and found 16068 isoforms (4634 upregulated isoforms and 11434 downregulated isoforms) corresponding to 8989 differentially expressed genes (2494 upregulated genes and 6495 downregulated genes).

Furthermore, volcano plots and MA plot views revealed distinct differences between the groups (control *vs*. Bam>*CG6015* RNAi; or Control *vs*. Bam>*CG6015* RNAi; Δ86/+) (Fig. 4b–c and Supplementary Fig. S2a–b). Importantly, circular heatmaps displayed the integrity distributions of the expression values and fold changes for the differentially expressed genes in testes (Fig. 4d and Supplementary Fig. S2c). When the altered transcripts were compared among the three groups (control, Bam>*CG6015* RNAi, and Bam>*CG6015* RNAi; Δ86/+), 7543 overlaps were identified, leading us to conclude that CG6015-mediated transcriptional regulation was important for male differentiation in testes (Fig. 4e).

To further analyze the biological events, gene ontology (GO) analysis was performed for the differentially expressed genes induced by spermatogonia *CG6015* RNAi. Interestingly, *CG6015* RNAi-mediated differentially expressed genes were mainly enriched in male gamete generation, spermatogenesis, spermatid differentiation, sperm motility, axoneme assembly, and protein localization to mitochondrion (Fig. 4f and Supplementary Fig. S2d). Moreover, examination of the downregulated differentially expressed genes revealed enrichments that were involved in spermatogenesis and spermatid differentiation, while the upregulated differentially expressed genes mainly participated in metabolic process (Supplementary Fig. S3). Taken together, the transcriptional profile analysis demonstrated that spermatogonia-derived CG6015 regulated spermatogenesis, spermatid differentiation, and metabolic processes.

### CG6015-mediated signaling pathways during spermatogonia TA-divisions

**Fig. 5:**
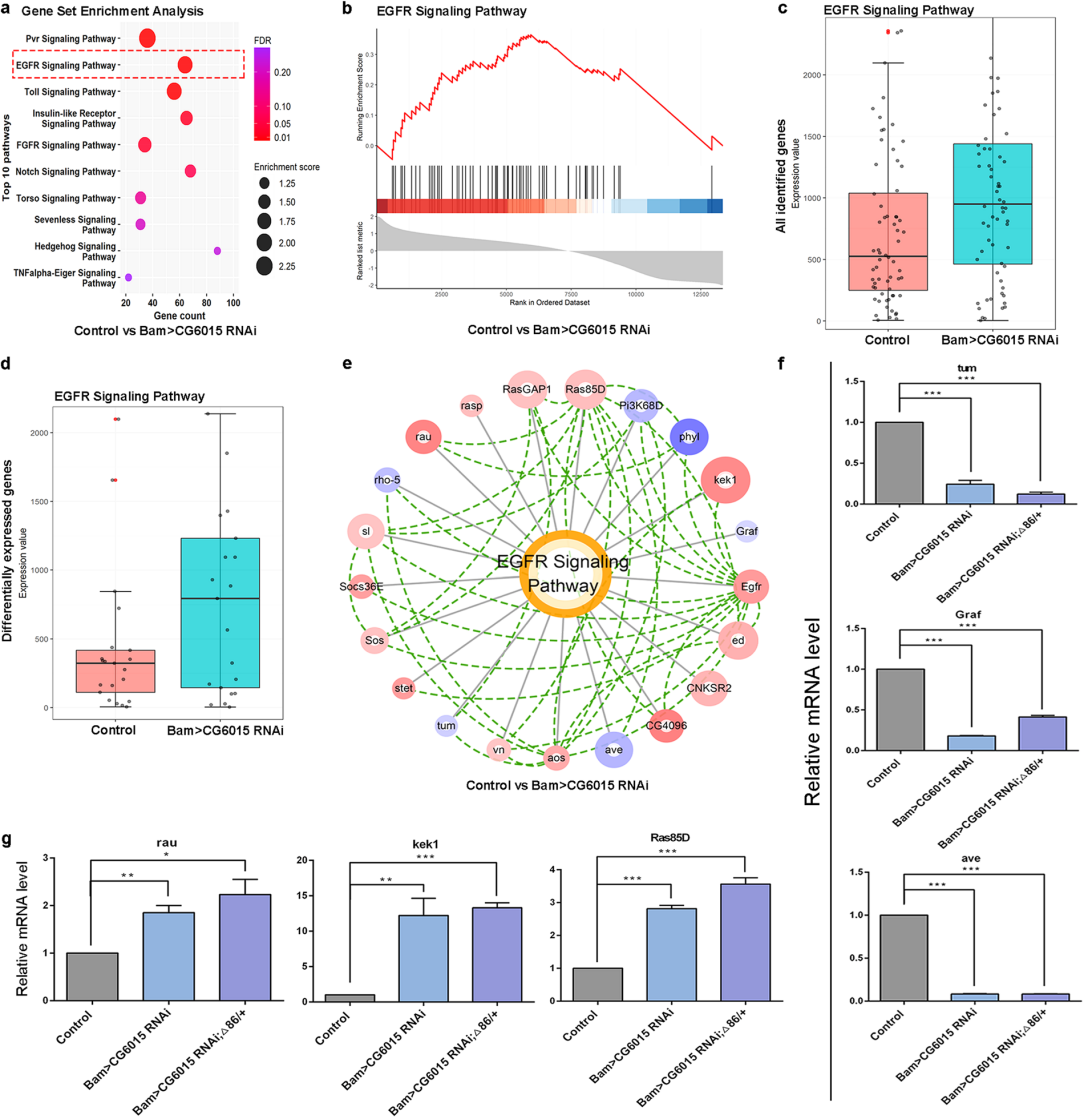
Inferred signaling pathways and CG6015-mediated regulators during spermatogonia TA-divisions. **a** Dot plots of the top 10 enriched pathways by GSEA from the comparisons of the control vs. Bam>*CG6015* RNAi testes. Color scale and dot size represent FDR value and enrichment score, respectively. **b** Enrichment plot for the EGFR pathway from the comparisons of the control *vs*. Bam>*CG6015* RNAi testes. Enrichment plot showing the distribution of the enrichment score, leading genes, and ranked list metric. **c** Box plots of EGFR signaling for all identified genes from the comparisons of the control *vs*. Bam>*CG6015* RNAi testes. **d** Box plots of EGFR signaling for differentially expressed genes from the comparisons of the control *vs*. Bam>*CG6015* RNAi testes. **e** Expression-interaction network of the EGFR signaling pathway from the comparisons of the control *vs*. Bam>*CG6015* RNAi testes. Blue and red scales represented low and high expression, respectively, while the circle size is proportional to the statistical significance (-logFDR). Green lines represented protein interactions. **f** Relative mRNA level of representative down-regulated genes in the control, Bam>*CG6015* RNAi and Bam>*CG6015* RNAi; Δ86/+ testes. **g** Relative mRNA levels of representative upregulated genes in the control, Bam>*CG6015* RNAi and Bam>*CG6015* RNAi; Δ86/+ testes. *P < 0.05, **P < 0.01, ***P < 0.001.

To explore key regulators and identify regulatory signaling pathways during spermatogonia differentiation, we performed pathway enrichment using Gene Set Enrichment Analysis (GSEA) of the global expression transcriptome profiles (Supplementary Tables S5–S6). Interestingly, we identified that the EGFR signaling pathway was enriched in the CG6015-mediated regulatory network in testes (Fig. 5a, b and Supplementary Fig. S4a–b). Moreover, box plot analysis of all identified genes and differentially expressed genes revealed that EGFR signaling was activated in *CG6015* RNAi or *CG6015* RNAi; Δ86/+ testes when compared with that in the controls (Fig. 5c, d and Supplementary Fig. S4c, d). Protein-protein interaction analysis further identified key modules of the EGFR signaling pathway with expression values and enrichments during spermatogonia TA-divisions (Fig. 5e and Supplementary Fig. S4e). We next verified the relative mRNA levels of representative regulators for the EGFR signaling pathway, and found that *tumbleweed* (*tum*), *GTPase regulator associated with FAK* (*Graf*), and *aveugle* (*ave*) were downregulated (Fig. 5f) while *rau*, *kekkon 1* (*kek1*), and *Ras oncogene at 85D* (*Ras85D*) were upregulated (Fig. 5g) in Bam>*CG6015* RNAi and Bam>*CG6015* RNAi; Δ86/+ testes when compared with those in the controls. This qRT-PCR verification was basically consistent with transcriptome profiling data. These data we provided clues to the relationship between CG6015 and the EGFR signaling pathway during spermatogonia TA-divisions in *Drosophila* testes.

### Spermatogonia derived CG6015 was essential for inhibiting germline dpERK

**Fig. 6:**
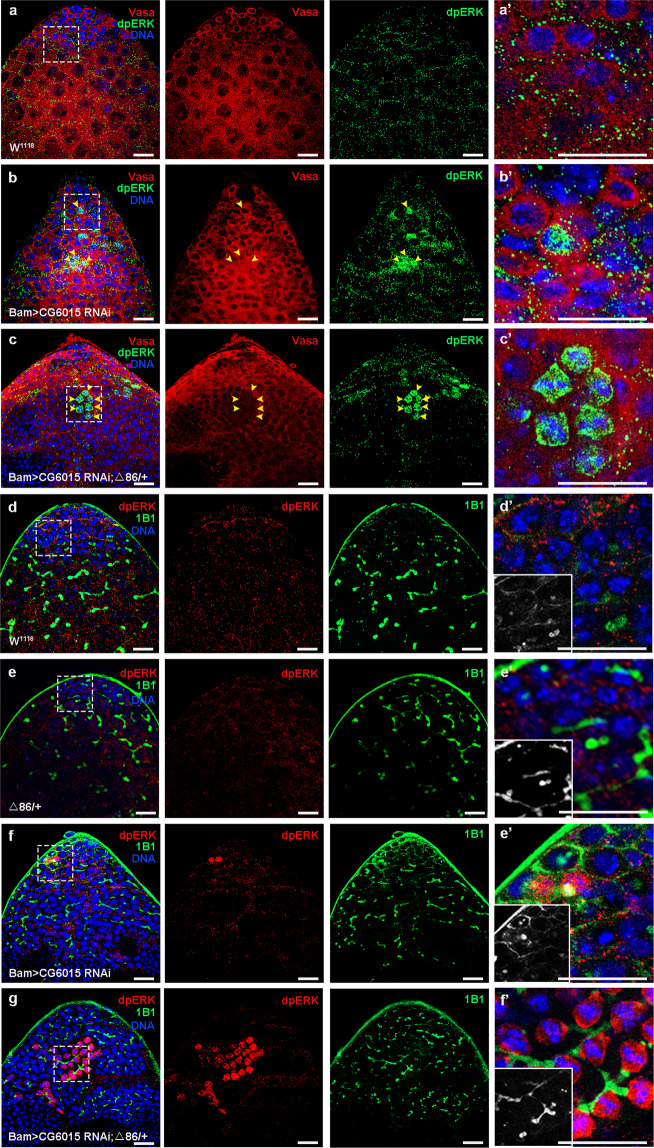
Reduction of CG6015 in spermatogonia upregulated dpERK in GSC-like cells. **a**–**c** Immunostaining of dpERK (green) and Vasa (red) at the apex of W^1118^, Bam>*CG6015* RNAi, and Bam>*CG6015* RNAi; Δ86/+ testes. Representative germline dpERK signals are shown by using yellow arrowheads. **a’**–**c’** Enlargements of the immunostaining of dpERK and Vasa signals. **d**–**g** Immunostaining of dpERK (red) and 1B1 (green) at the apex of W^1118^, Δ86/+, Bam>*CG6015* RNAi and Bam>*CG6015* RNAi; Δ86/+ testes. **d’**–**g’** Enlargements of immunostaining of dpERK and 1B1 signals. DNA was stained with Hoechst (blue). Scale bars: 20 µm.

To exam the effect of the EGFR signaling pathway in spermatogonia in *Drosophila* testes, we next stained with dpERK, which is a positive effector of the EGFR signaling pathway and was mainly expressed in CySCs^36^. Germline dpERK signals were upregulated in spermatogonia in Bam>*CG6015* RNAi testes, and this phenotype was dramatically enhanced in Bam>*CG6015* RNAi; Δ86/+ testes (Fig. 6a–c). Furthermore, co-staining of dpERK and 1B1 demonstrated that a series of germline dpERK signals, mediated by *CG6015* RNAi, were tightly integrated through fusomes in testes, indicating that these germ cells originated from the same GSCs (Fig. 6d–g). To further confirm the consistency in testes, we quantified the number of fusomes with germline dpERK signals, and discovered that there were 0.8333 ± 0.2748 relevant fusomes in Bam>*CG6015* RNAi testes (*n* = 6) and 7.286 ± 1.104 relevant fusomes in Bam>*CG6015* RNAi; Δ86/+ testes (*n* = 14) when compared with control testes (no relevant fusome, *n* = 12). Therefore, we speculated that *CG6015* RNAi in spermatogonia led to differentiation defects and subsequently activated dpERK signaling in GSC-like cells.

### Downstream of raf1 (Dsor1) was required for spermatogonia differentiation and regulated germline dpERK in *Drosophila* testes

**Fig. 7:**
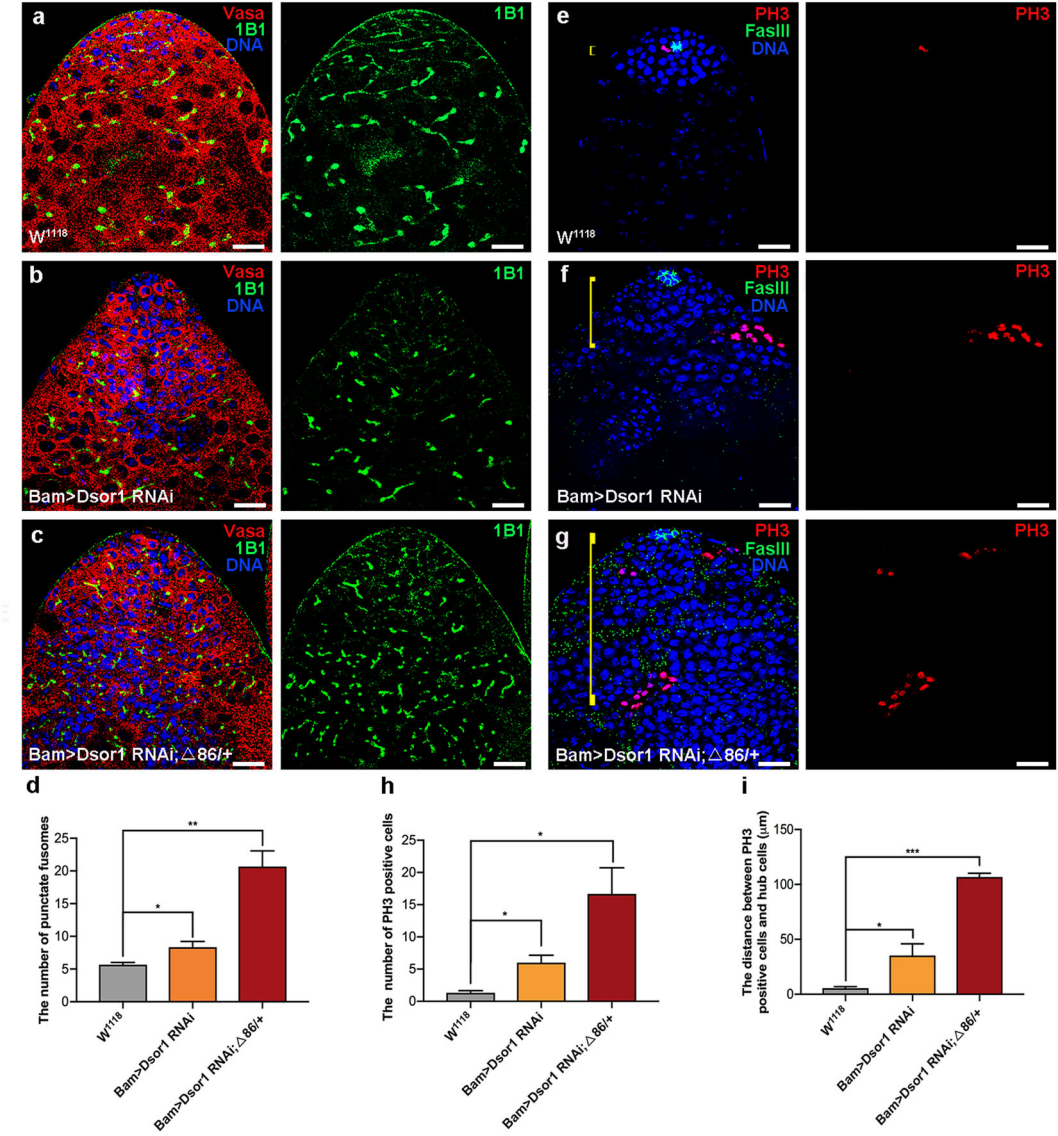
Downregulation of Dsor1 in spermatogonia resulted in germline differentiation defects. **a–c** Immunostaining of Vasa (red) and 1B1 (green) at the apex of W^1118^, Bam>*Dsor1* RNAi, and Bam>*Dsor1* RNAi; Δ86/+ testes. **d** The number of punctate fusomes in W^1118^ (*n* = 3), Bam>*Dsor1* RNAi (*n* = 3), and Bam>*Dsor1* RNAi; Δ86/+ (*n* = 3) testes. **e–g** Immunostaining of PH3 (red) and FasIII (green) at the apex of W^1118^, Bam>*Dsor1* RNAi, and Bam>*Dsor1* RNAi; Δ86/+ testes. Yellow lines represent the distance between PH3-positive cells and hub cells. **h** The number of PH3-positive cells in W^1118^ (*n* = 3), Bam>*Dsor1* RNAi (*n* = 3), and Bam>*Dsor1* RNAi; Δ86/+ (*n* = 3) testes. **i** The distance between PH3-positive cells and hub cells in W^1118^ (*n* = 3), Bam>*Dsor1* RNAi (*n* = 3), and Bam>*Dsor1* RNAi; Δ86/+ (*n* = 3) testes. DNA was stained with Hoechst (blue). *P < 0.05, **P < 0.01, ***P < 0.001. Scale bars: 20 µm.

Dsor1 is also a critical downstream mediator of the EGFR signaling pathway, and could phosphorylate ERK^37^. We next investigated the function of Dsor1 in spermatogonia in *Drosophila* testes. The results showed that downregulation of Dsor1 driven by Bam-Gal4 led to germ cell differentiation defects and induced GSC-like cysts (Fig. 7a, b). Moreover, germline differentiation defects were enhanced by heterozygous mutation of *bam* (Δ86/+) in Bam>*Dsor1* RNAi testes (Figs. 7a and c). Meanwhile, the number of punctate fusomes was increased in both Bam>*Dsor1* RNAi and Bam>*Dsor1* RNAi; Δ86/+ testes (Fig. 7d). Furthermore, the number of PH3-positive cells and the distance between PH3-positive cells and hub cells were dramatically increased in Bam>*Dsor1* RNAi and Bam>*Dsor1* RNAi; Δ86/+ testes, when compared with those in control testes (Fig. 7e–i). We next stained with Vasa and dpERK to estimate germline dpERK signals in *Dsor1* RNAi testes. Notably, reduction of *Dsor1* in spermatogonia activated germline dpERK signals in Bam>*Dsor1* RNAi and Bam>*Dsor1* RNAi; Δ86/+ testes (Supplementary Fig. S5). Our data demonstrated that Dsor1 mimicked the phenotype of CG6015 in spermatogonia and regulated the abundance of dpERK in *Drosophila* testes.

## Discussion

Spermatogonia TA-divisions control germline homeostasis and instruct the precise transition from proliferation to meiotic differentiation^5^. Bam, a key differentiation factor, is specifically expressed among differentiated spermatogonia, and ectopic expression of Bam promoted germline differentiation^38^. Furthermore, evidence showed that three RNA binding proteins (Tut, Bam, and Bgcn) formed a physical and functional complex to regulate spermatogonia TA-divisions in *Drosophila*, and were necessary to repress mei-P26 to promote GSC differentiation^26^.

In *Drosophila* testes, CySCs and hub cells form niche signals and provide an instructive microenvironment for GSC self-renewal^39^. Meanwhile, CySCs could provide somatic signals which trigger GSC differentiation, and deficiency of CySCs and cyst cells resulted in failure to exit the TA-divisions and inhibited germline differentiation^35^. In contrast to the niche signals, we investigated roles of CG6015 during spermatogonia TA-divisions, and found that CG6015 was essential for germline differentiation via cell autonomous effects in spermatogonia. Loss of function of CG6015 in spermatogonia led to extra accumulation of undifferentiated germ cells that could not differentiate into gametes. We also explored the regulatory network using RNA-seq to clarify the potential mechanisms of EGFR signaling in CG6015-mediated differentiation defects.

Elaborate modifications of the spermatid components are key steps for spermiogenesis in *Drosophila*. GSCs divide into spermatogonia and undergo four rounds of mitosis, producing 16 spermatocytes. Then, they enter into meiosis and result in 64 spermatids which were connected by cytoplasmic bridges and encapsulated by two somatic cyst cells^40^. After meiosis within a cyst, the nuclei of 64 elongated spermatozoa are condensed into a sperm bundle. Via spermatid individualization, mature swimming sperm are finally released^41^. Actually, mitochondria also play critical roles throughout the whole process of spermiogenesis. In *Drosophila*, mitochondria are distributed in the cytoplasm during early stage of spermatogenesis. By the end of meiosis, mitochondria gradually aggregate and fuse into a two-part spherical structure, named ‘Nebenkern’. During spermatid elongation, the mitochondrial derivative-nebenkern starts to lengthen parallel to the axis at both ends^42,43^. In this study, we discovered that the structure of clusters of elongated spermatids was loose in *CG6015* RNAi testis when compared with the controls, indicating that CG6015 was required to keep spermatids bundled tightly. Moreover, the number of elongated spermatid clusters was significantly reduced in *CG6015* RNAi testis. Importantly, differentially expressed genes, which were mediated by *CG6015* RNAi in *Drosophila* testes, were enriched in protein localization to mitochondrion. These results indicated that spermatogonia-derived CG6015 may function in mitochondrion to maintain spermatids elongation and development. Although mitochondrial behavior during spermiogenesis is fairly well understood, the molecular mechanisms between CG6015 and mitochondrial genes remain further investigations.

The fusomes branches throughout germ cell differentiation within a cyst, synchronizing the behavior of germ cells^44^. Previous study has illustrated the visualization of dot-like fusomes, also named punctate fusomes, at the apical tip of testes connecting GSCs and their daughter GBs, as well as the branched network that strings spermatogonia or spermatocytes together within a cyst^45^. Therefore, the accumulation of punctate fusomes far away from hub cells implies over proliferation or differentiation defects in *Drosophila* testes. As previous study reported, PH3 staining is used to track mitosis. Usually, 2-, 4-, 8- and 16- cell cysts appeared PH3-labeled signals in wild type testes, indicating that germ cells within a cyst always divide together^21^. Here, PH3-positive cells in *CG6015* RNAi testes were dramatically increased, indicating the delayed mitosis mediated by CG6015. Moreover, clusters of PH3-positive cells in *CG6015* RNAi testes were far away from hub cells, suggesting that loss of CG6015 in spermatogonia substantially desynchronized the mitosis of germ cells. Altogether, these data displayed the significant roles of CG6015 in regulating germ cells divisions during spermatogonia TA-divisions.

Previous reports showed that downstream effectors of EGFR signaling function through cellular extensions of somatic cyst cells to organize microenvironments for germ cell differentiation in *Drosophila* testes^46^. Evidence showed that the *stet* gene, whose encoded protein could catalyze proteolytic cleavage of spi within the Golgi, was required for male fertility and encapsulation of germ cells to promote differentiation via EGFR signaling on somatic cyst cells^47^. The Raf oncogene (Raf), which acts downstream of Ras, activates the MEK/ERK pathway to regulate cell proliferation and differentiation^48–50^. Moreover, Raf deficiency disturbed cyst progenitor cell identity and somatic signaling, and further produced excess undifferentiated germ cells in *Drosophila* testes^51^. A previous study showed that activation of EGFR signaling in somatic cells repressed Dally, thereby affecting the movement and stability of the Dpp protein, leading to germline differentiation^52^. This evidence indicated that somatic EGFR signaling plays key roles in normal germ cell differentiation and forms crucial associations between soma and germline cells.

dpERK, a key EGFR downstream target, was enriched in cyst cells, but was not expressed in germline cells in *Drosophila* testes^53^. However, the roles of germline EGFR signaling are rarely studied and discussed, and the mechanism of germline EGFR signaling is unknown. In the present study, we used dpERK to assess the activation of EGFR signaling. We further determined the relationship between CG6015 and EGFR signaling during spermatogonia TA-divisions in *Drosophila* testes. Interestingly, we observed the existence of germline dpERK signals, and CG6015 mediated germline differentiation defects by directly activating germline dpERK in GSC-like cysts. In addition to the previously reported roles for the formation of CySCs, EGFR signaling also displayed dose- dependent effects on differentiation. Interestingly, a low level of EGFR signaling was required for spermatogonia TA-divisions, and a high level of EGFR signaling promoted germline differentiation in *Drosophila* testes^54^. Based on the above evidence, we speculated that ectopic production of germline dpERK among undifferentiated GSC-like cells might be an instinctive cellular response to promote differentiation.

In summary, we explored roles of CG6015 during spermatogonia TA-divisions, and revealed the relationship between CG6015 and EGFR signaling in *Drosophila* testes. Our data strongly suggested that CG6015 regulates germline differentiation via germline dpERK signals. This study extends our understanding of spermatogonia TA-divisions and germline homeostasis, and could be used as the basis to investigate crosstalk between somatic and germline cells.

## Materials and methods

### Fly strains

All flies were raised on standard cornmeal molasses agar medium at 25 °C and in a relative humidity of 40–60%. UAS-RNAi transgenic flies were obtained from the TsingHua Fly Center (THFC) and detailed information was as follows: UAS-*CG6015* RNAi (#THU1409), UAS-*Dsor1* RNAi (#THU0677). Bam-Gal4; Δ86/+ and Bam-GFP; Δ86/+ lines were gifts from DH Chen (Institute of zoology, Chinese Academy of Sciences, Beijing, China).

### Fly crosses

Two to three-day-old flies were used in this study. Male flies of the Bam-Gal4; Δ86/+ line were randomly chosen to cross with transgenic UAS-RNAi virgins and raised at 25 °C until hatching. F1 males with a specific genotype (Bam > RNAi or Bam>RNAi; Δ86/+) were selected for the further analysis. The W^1118^ and Δ86/+ lines were used as controls.

### Quantitative real-time reverse transcription PCR

Quantitative real-time reverse transcription PCR (qRT-PCR) was performed according to the manufacturer’s protocol. Total RNA was extracted using TRIzol Reagent (15596026, Invitrogen, Waltham, MA, USA). Reverse transcription was performed using HiScript III 1st Strand cDNA Synthesis Kit (R312-01, Vazyme, Jiangsu, China). TB Green Premix Ex Taq II (RR820, Takara, Shiga, Japan) was used to carry out the qPCR step. The primers used in this study are shown in Supplementary Table S7.

### Immunostaining and antibodies

Fly testes were dissected in 1× phosphate-buffered saline (PBS), fixed for 30 min in 4% paraformaldehyde, washed with 0.3% PBS-Triton X-100 (PBST) three times, and blocked in 5% bovine serum albumin for 30 min. Testes were incubated with primary antibodies at room temperature for 1 h. Then, testes were washed three times in 0.3% PBST and incubated with secondary antibodies at room temperature for 1 h in the dark. After washing three times again with 0.3% PBST, testes were stained with Hoechst-33342 (1.0 mg/ml, C0031, Solarbio, Beijing, China) for 5 min before mounting. The primary antibodies used were as follows: rat anti-Vasa [Developmental Studies Hybridoma Bank (DSHB), University of Iowa, Dept of Biology, Iowa City, IA, USA; 1:20], mouse anti-1B1 (DSHB, 1:50), mouse anti-FasIII (DSHB, 1:50), rat anti-Zfh1 (a gift from Prof. Chao Tong, 1:1000), mouse anti-Eya (DSHB, 1:30), rabbit anti-PH3 [#53348, Cell Signaling Technology (CST), Danvers, MA, USA; 1:1000], rabbit anti-dpERK (#4370, CST, 1:200). Secondary antibodies conjugated with A488, Cy3, or A647 (Jackson ImmunoResearch Laboratories, West Grove, PA, USA) were diluted at 1:1000.

### Images acquisition and analysis

The images for *Drosophila* testes were obtained by microscope and analyzed by Image J software according to usage introductions. For the measurement of distance between hub cells and PH3 positive cells, Image J was used to draw a line between hub cells and the farthest PH3 positive cells. The measurement results would be given in the form of pixel values. Necessary parameters are required to been set in image J for transforming the measurement data from pixel values to physical dimensions.

### Preparation and RNA-Seq

We used two to three-day-old flies as controls, and Bam>CG6015 RNAi and Bam>CG6015 RNAi; Δ86/+ genotypes in this study. Testes from 50 male flies of each genotype were dissected in cold PBS. Total RNA was prepared from the isolated testes using TRIzol (15596026, Invitrogen), following the recommendations of the manufacturer. The concentration and purity of the total RNA were detected using a Nanodrop 2000 instrument (Nanodrop Technologies, Wilmington, DE, USA), and the integrity of the RNA was detected by gel electrophoresis, and the RNA integrity (RIN) values were measured using an Agilent 2100 instrument (Agilent Technologies, Santa Clara, CA, USA). Indexed RNA-Seq libraries were prepared from 800 ng of total RNA using the TruSeq RNA Library Prep Kit v2 (Illumina, San Diego, CA, USA)) following the manufacturer’s protocol. This experiment included purification of poly (A) mRNA with oligo-dT magnetic beads, RNA fragmentation, synthesis of double-stranded cDNA using SuperScript II reverse transcriptase (Invitrogen), ligation of indexed Illumina adapters, and amplification using limited-cycle PCR. Sequencing libraries were validated by capillary electrophoresis using a Bioanalyzer 2100 instrument (Agilent Technologies). The DNB (DNA nano ball) was prepared after the libraries were tested for qualification, and then loaded into the sequencing chip. Sequencing was performed using a high-throughput sequencer (MGIseq2000, MGI, Shenzhen, China).

### Bioinformatic analysis of RNA-seq data

Reads were aligned to the *Drosophila* melanogaster reference genome in NCBI’s assembly resource (www.ncbi.nlm.nih.gov/assembly/) using the Bowtie package with Hierarchical Indexing for Spliced Alignment of Transcripts (HISAT) comparison software^54^. The transcript abundances were determined using Fragments Per Kilobase per Million mapped reads (FPKM) values with the RSEM tool^55^. Differentially expressed genes were identified using criteria of a an FDR < 0.05 and log2FC > 1 (or log2F < −1). Volcano plot views were obtained to display visually the distribution of FDR and FC values of differentially expressed genes between two groups. The number of differentially expressed genes among multiple groups was analyzed using Venn diagrams. Gene ontology (GO) enrichment analysis was performed using the hyper-geometric distribution for total, downregulated, and upregulated differentially expressed genes, and graphs were shown with GO terms, rich factor, gene number, and –log10 (Q value). For the circular heatmap, all differentially expressed genes were normalized by the Z-score and clustered using MeV (version 4.9.0)^56^. The circular heatmap was generated using the circlize package (version 0.4.11) in R software^57^. For GSEA, among all the identified genes, those with extremely low expression values (below 5) were filtered out, and all reserved genes were ranked in descending order according to their degree of change. The reference gene sets (known signaling pathways) were extracted from the gene group annotations in FlyBase^58^. GSEA was performed using the GSEA function embedded in the cluster Profiler package (version 3.18.0) in the R software^59^. An FDR of 0.05 was set as the cutoff for a statistically significant enrichment. Box plots were generated by using the ggplot2 package in the R software. The protein-protein interactions were obtained from the STRING database using a medium score^60^. The complex expression-interaction network was generated using Cytoscape (version 3.9.0)^61^.

### Statistical analysis for functional experiments

All the functional experiments conducted in this study were repeated at least three times. The quantitative results were presented as means ± standard error of the mean (SEM) and evaluated for statistical differences using Student’s *t*-test and one-way analysis of variance (ANOVA) using GraphPad software (GraphPad Inc., La Jolla, CA, USA). *P < 0.05; **P < 0.01; ***P < 0.001.

## Supplementary information

SUPPLEMENTAL FILE

Table S1

Table S2

Table S3

Table S4

Table S5

Table S6

Table S7
