# Supplementary material for: CG6015 controls spermatogonia transit-amplifying divisions by epidermal growth factor receptor signaling in Drosophila testes
Source: Cell Death Dis. 2021 May 14;12(5):491. doi: 10.1038/s41419-021-03783-9 (PMC8121936; doi:10.1038/s41419-021-03783-9)
Supplement: Supplementary file 1 — SUPPLEMENTAL FILE [file 41419_2021_3783_MOESM1_ESM.doc]

**
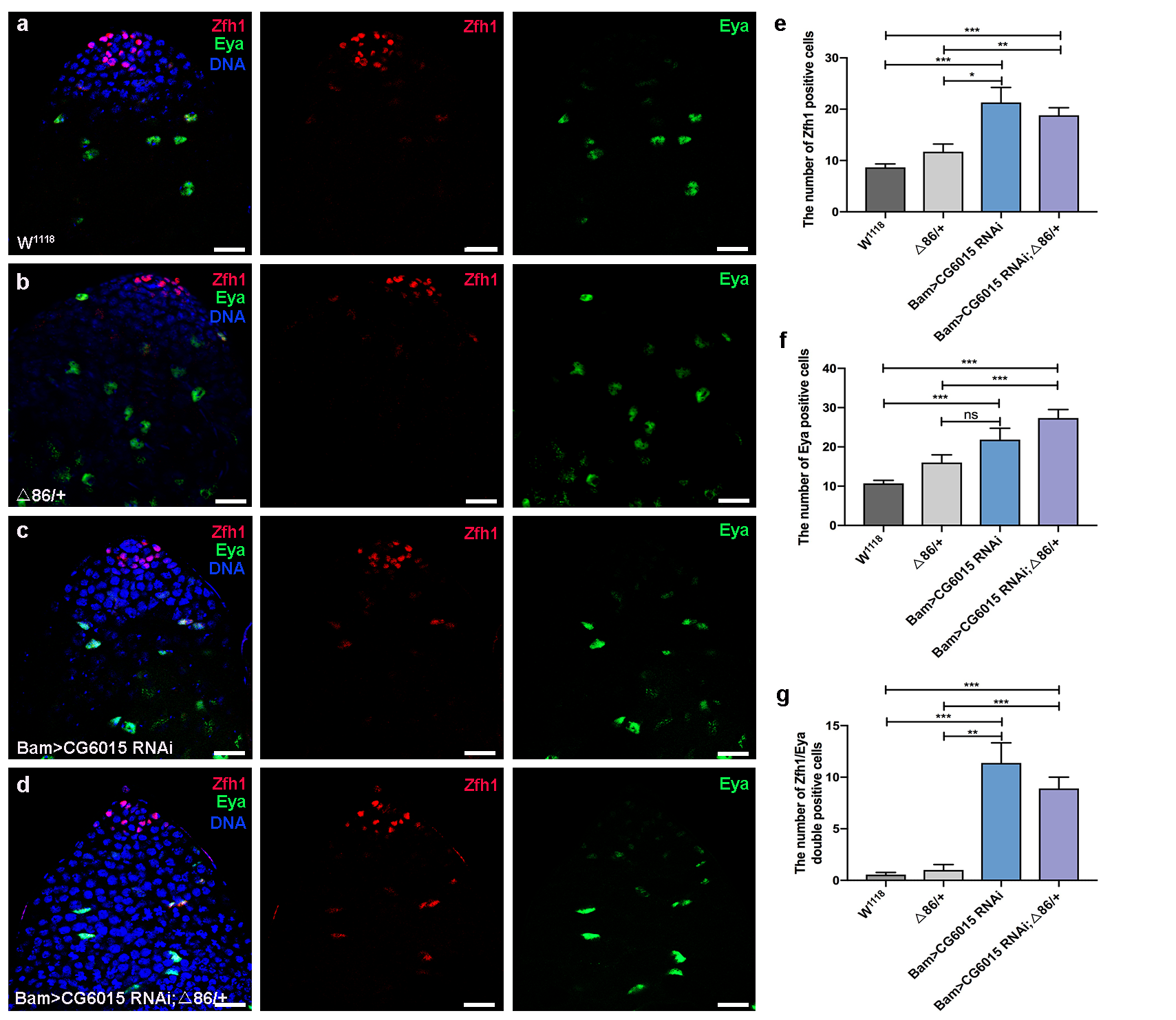
**

**Fig. S1 The differentiation of cyst cells was disrupted by reduction of CG6015 in spermatogonia.** **a-d** Immunostaining of Zfh1 (red) and Eya (green) at the apex of W^1118^, Δ86/+, Bam>*CG6015* RNAi and Bam>*CG6015* RNAi; Δ86/+ testes. **e** The number of Zfh1 positive cells in W^1118^ (n=20), Δ86/+ (n=7), Bam>*CG6015* RNAi (n=13) and Bam>*CG6015* RNAi; Δ86/+ (n=11) testes. **f** The number of Eya positive cells in W^1118^ (n=20), Δ86/+ (n=7), Bam>*CG6015* RNAi (n=13) and Bam>*CG6015* RNAi; Δ86/+ (n=11) testes. **g** The number of Zfh1/Eya double positive cells in W^1118^ (n=20), Δ86/+ (n=7), Bam>*CG6015* RNAi (n=13) and Bam>*CG6015* RNAi; Δ86/+ (n=11) testes. * *P* < 0.05, ** *P* < 0.01, *** *P* < 0.001, ns. not significant. Scale bar: 20 μm.

**
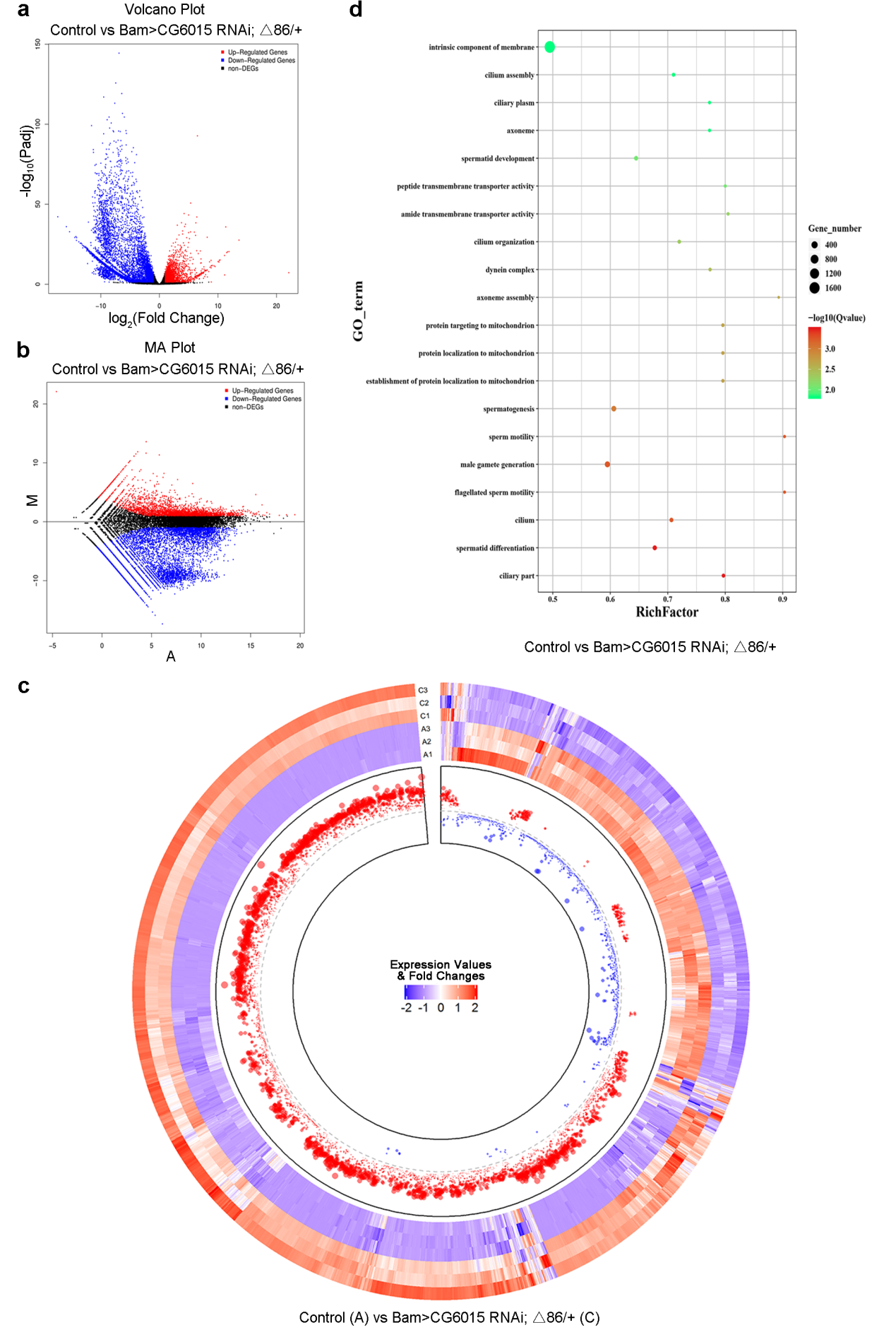
**

**Fig. S2 Bioinformatics analysis of CG6015 and Bam mediated spermatogonia TA‑divisions.** **a** Volcano plots based on –log_10_Padj and log_2_FC from the comparison of the control and Bam>*CG6015* RNAi; Δ86/+ groups. **b** MA plots from the comparison of the control and Bam>*CG6015* RNAi; Δ86/+ groups. **c** Circular heatmap of differentially expressed genes from the comparison of the control and Bam>*CG6015* RNAi; Δ86/+ groups. The outer and inner tracks represent the expression values and fold changes, respectively. For both the expression value and fold change, blue and red scales represent low and high expression, respectively. **d** GO analysis of the sets of differentially expressed genes from the comparison of the control and Bam>*CG6015* RNAi; Δ86/+ groups.

**
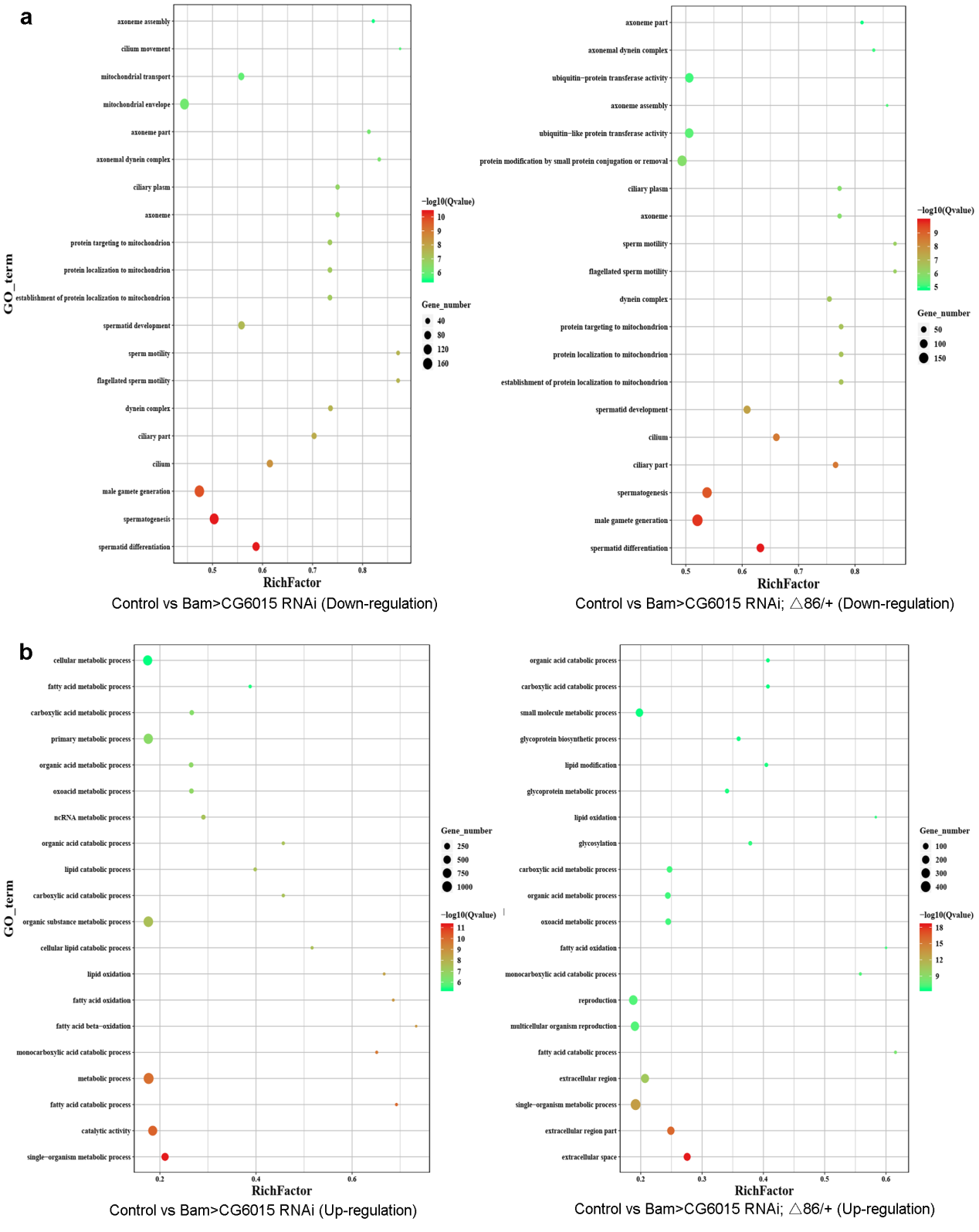
**

**Fig. S3** **GO analysis of differentially expressed genes in subgroups.** **a** GO analysis of down-regulated differentially expressed genes from the comparisons of the control *vs.* Bam>*CG6015* RNAi (or control *vs.* Bam>*CG6015* RNAi; Δ86/+) testes. **b** GO analysis of up-regulated differentially expressed genes from the comparisons of the control *vs*. Bam>*CG6015* RNAi (or control *vs.* Bam>*CG6015* RNAi; Δ86/+) testes.

**
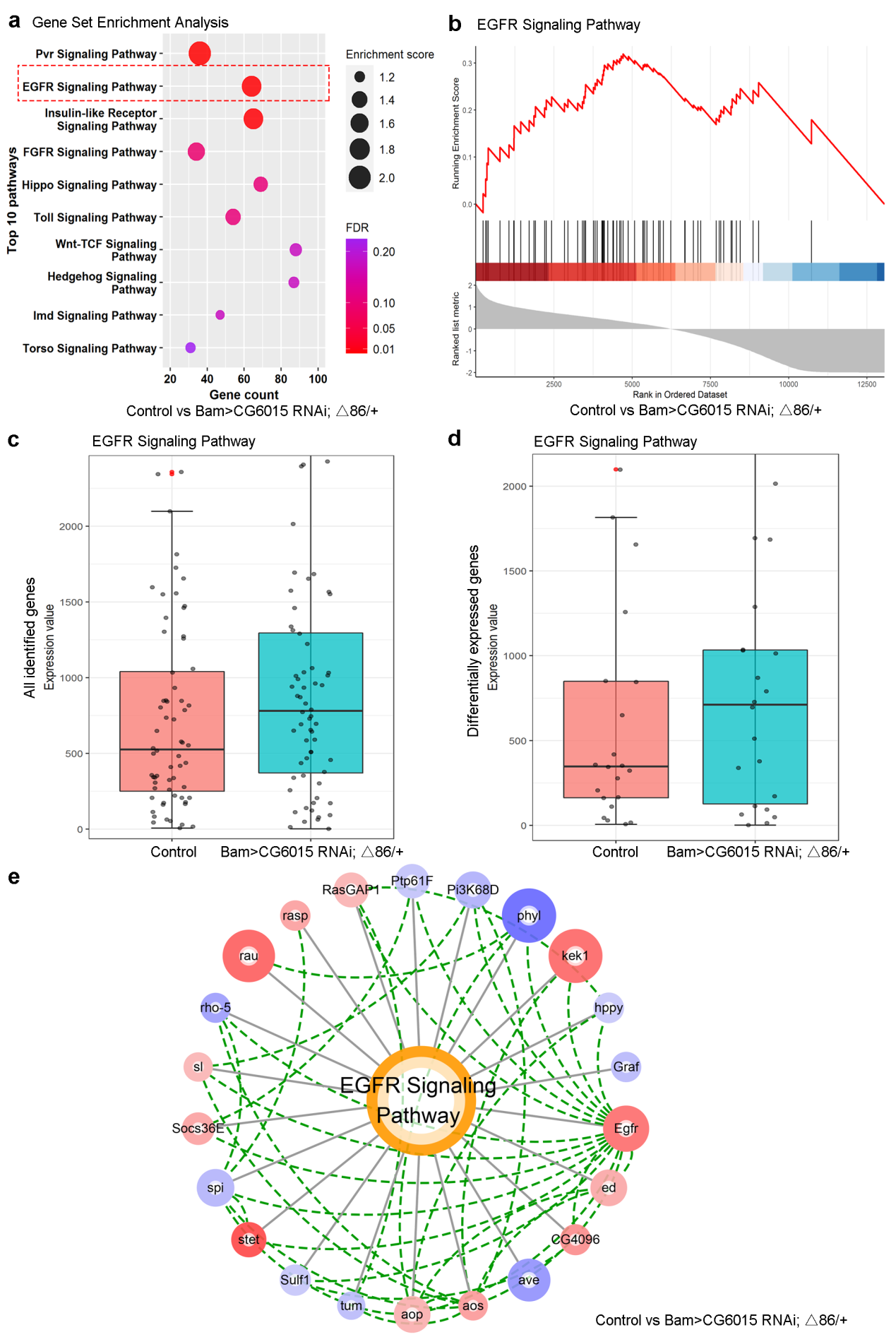
**

**Fig. S4 Inferred signaling pathway analysis of CG6015 and Bam-mediated spermatogonia TA-divisions. a** Dot plots of the top 10 enriched pathways by GSEA for the comparisons of the control *vs*. Bam>*CG6015* RNAi; Δ86/+ testes. The color scale and dot size represent the FDR value and enrichment score, respectively. **b** Enrichment plot for the EGFR pathway from the comparisons of the control *vs.* Bam>*CG6015* RNAi; Δ86/+ testes. Enrichment plot showing the distribution of the enrichment score, leading genes, and ranked list metric. **c** Box plots of EGFR signaling for all identified genes from the comparisons of the control *vs.* Bam>*CG6015* RNAi; Δ86/+ testes. **d** Box plots of EGFR signaling for differentially expressed genes from the comparisons of the control *vs.* Bam>*CG6015* RNAi; Δ86/+ testes. **e** Expression-interaction network of the EGFR signaling pathway from the comparisons of the control *vs.* Bam>*CG6015* RNAi; Δ86/+ testes. Blue and red scales represent low and high expression, respectively, while the circle size is proportional to the statistical significance (-logFDR). Green lines represented protein interactions.


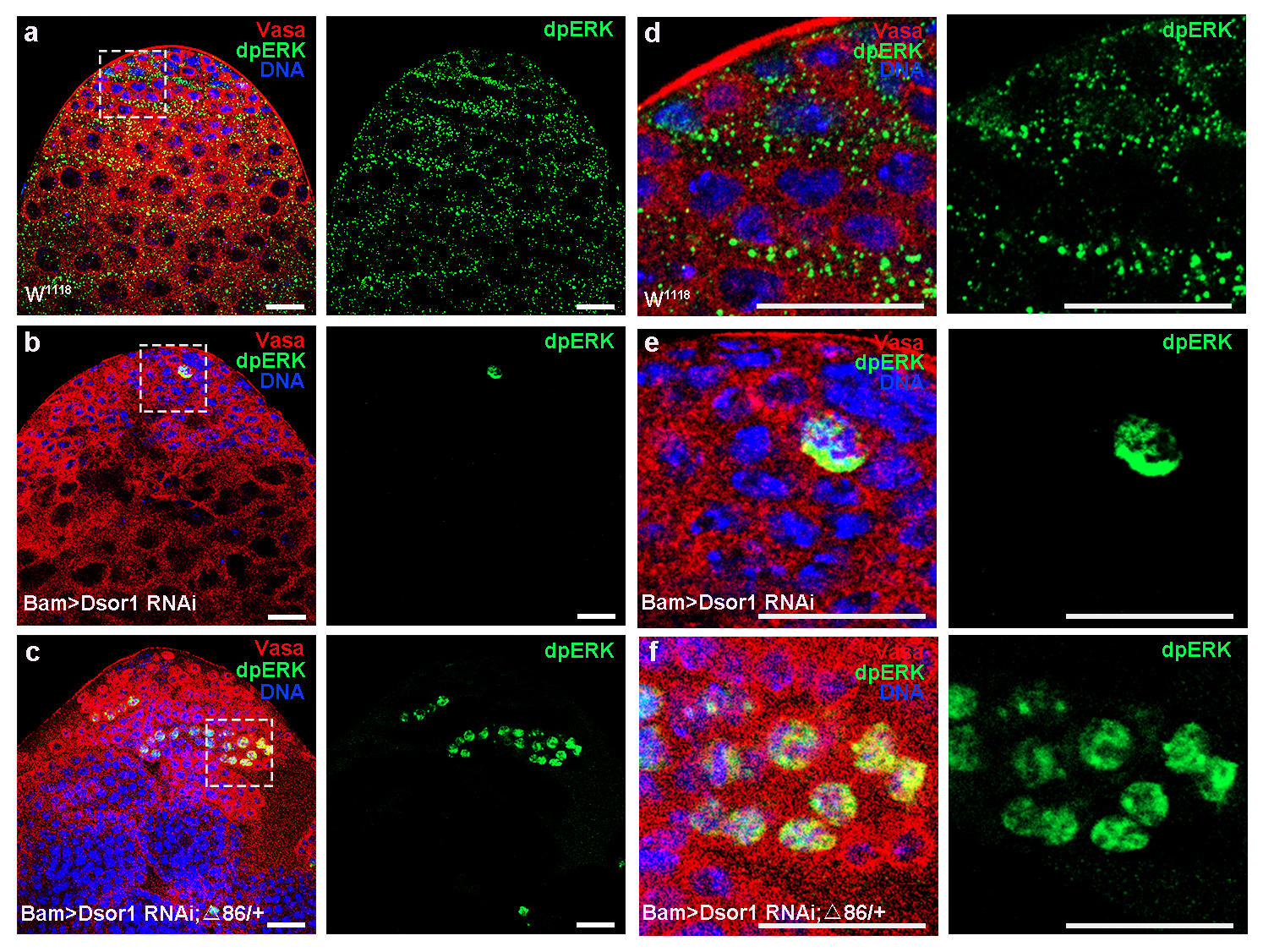


**Fig. S5 Downregulation of Dsor1 in spermatogonia led to altered abundance of germline dpERK.** **a-c** Immunostaining of Vasa (red) and dpERK (green) at the apex of W^1118^, Bam>*Dsor1* RNAi and Bam>*Dsor1* RNAi; Δ86/+ testes. **d-f** Enlargements of the immunostaining of dpERK and Vasa signals. DNA was stained with Hoechst (blue). Scale bars: 20 µm.
