# Supplementary material for: CG6015 controls spermatogonia transit-amplifying divisions by epidermal growth factor receptor signaling in Drosophila testes
Source: Cell Death Dis. 2021 May 14;12(5):491. doi: 10.1038/s41419-021-03783-9 (PMC8121936; doi:10.1038/s41419-021-03783-9)
Supplement: Supplementary file 8 — Table S7 [file 41419_2021_3783_MOESM8_ESM.docx]

**Table S7. All primer sequences used in this study.**

| **Gene** | **Forward (5'-3')** | **Reverse (5'-3')** |
| --- | --- | --- |
| GAPDH | GTGGTGAACGGCCAGAAGAT | GCCTTGTCAATGGTGGTGAA |
| CG6015 | GCCTGCCAGTCGTTAGACAA | GCGTAGCCAGAGACCATGTG |
| tum | CAGTGTCAGAAACGCATACGG | CCTTGACACAACAGCACAGC |
| ave | ACCTGTGGACAGTTAGCGATG | CTTCCGGTTATATCGTGCTGG |
| rau | CGTACGCATACGCACCTTTG | AGCTGCGCTTACCTTCTAGC |
| Ras85D | ATTGCAATTACCGCACGGC | TGTGCGTGTATGGGCTTCTT |
| kek1 | ACGGATGGGAGGTAGCTCTT | ACGTTATGCGGGGAGTTGAG |
| Graf | GCTCCATTGTGGCCAATAGT | CTAACCGAGGGCAATGTTGT |
